# Editorial: Intra- and extra-environment and reproduction

**DOI:** 10.3389/fcell.2022.1020470

**Published:** 2022-11-04

**Authors:** Zhao-Jia Ge, Francesca Gioia Klinger, Teruko Taketo

**Affiliations:** ^1^ Key Laboratory of Animal Reproduction and Germplasm Enhancement in Universities of Shandong, College of Life Sciences, Institute of Reproductive Sciences, Qingdao Agricultural University, Qingdao, China; ^2^ Histology and Embryology, Saint Camillus International University of Health Sciences, Rome, Italy; ^3^ Department of Obstetrics and Gynecology, Department of Surgery, Department of Biology, McGill University Health Centre, McGill University, Montréal, QC, Canada

**Keywords:** environmental factors, oocyte, sperm, ovary, metabolic disorders, reproduction

## Introduction

Infertility is a challenging problem for people who desire to have children. Approximately 10–15% of reproductive-age couples are affected by infertility (Wasilewski et al., 2020; Saha et al., 2021). The causes of infertility are various, with approximately 30% for male factors, 30% for female factors, 30% for both partners, and 8–28% for unexplained reasons (Saha et al., 2021) (https://www.singlecare.com/blog/news/infertility-statistics/). In females, the most common causes of infertility are ovarian dysfunction (25–35%), tubal-related problems (20–25%), uterine pathology (15–30%), and unexplained reasons (20–30%) (Takasaki et al., 2018; Szamatowicz and Szamatowicz, 2020). In males, low sperm quality (35%) is the main cause of infertility (Odisho et al., 2014; Carson and Kallen, 2021). These causes of infertility can be attributed to other common health issues (Carson and Kallen, 2021). For example, metabolic disorders, such as obesity and diabetes, induce low oocyte quality, abnormal epigenetic modifications in oocytes and sperm, and impaired embryo development (Ou et al., 2019; Snider and Wood, 2019; Kusuyama et al., 2020). PCOS (polycystic ovarian syndrome), a common endocrine disease in reproductive-age women, leads to anovulation, low oocyte quality and fertilization rate, and subfertility/infertility (Risal et al., 2019; Kumariya et al., 2021). Exposure to phthalates, widely used in the manufacture of plastics, leads to premature ovarian failure by disrupting the reproductive endocrine functions (Lambrot et al., 2009; Lehraiki et al., 2009; Rajkumar et al., 2022). Pesticide residues are deleterious to ovarian function and oocytes (Biggs et al., 2008; Liu et al., 2021). Lifestyle factors such as smoking also contribute to infertility, (Esakky and Moley, 2016; Engel et al., 2021). However, the mechanisms of environmental factors underlying infertility are not yet fully understood. This Research Topic is focused on the intra- and extra-environmental factors affecting reproduction.

## Regulation of gametogenesis

In mammals, haploid germ cells are produced from the diploid precursor cells through meiosis (Larose et al., 2019). In females, meiosis is initiated in the fetal ovary, and the primary oocytes are arrested at Meiotic Prophase I, enclosed in primordial follicles, perinatally in mice. At puberty, a cohort of the primordial follicles are recruited in the growth phase, and the oocytes resume the meiotic cell cycle to go through the first meiosis division and become mature oocytes. Accurate chromosome segregation depends on many factors including chromosomal and ooplasmic components. For example, centromeres and telomeres are two crucial regions in chromosomes and play a key role in regulating chromosome segregation during oocyte meiosis (Meerdo et al., 2005; Kazemi and Taketo, 2021). Jeon and Oh report that the deletion of TRF1, a component of the telomeric protein complex, resulted in the dysfunction of the spindle-assembly checkpoint (SAC) and an increase in the aneuploidy rate in mouse oocytes. mRNA accumulation during oocyte growth/follicular development is crucial for oocyte competency and early embryo development (Ruebel et al., 2021). At the end of the growth phase of oocytes, when they are commonly referred to as germinal vesicles (GV), the oocyte ceases transcription and the mRNA accumulated is programmatically degraded during meiotic progression (Gindi et al., 2022). The poly(A) tail length at the 3′ end is important for mRNA stability in oocytes (Yang et al., 2020). The CCR4-NOT complex regulates mRNA degradation through deadenylation (shortening) of the poly(A) tail (Reyes and Ross, 2016). Epigenetic modification is another important factor that regulates mRNA stability in oocytes. For example, N6-methyladenosine (m6A) modification plays a key role in stabilizing the mRNA of oocytes and early embryos (Kasowitz et al., 2018). Another modification, N4-acetylcytidine (ac4C), of mRNA was found to regulate translation (Arango et al., 2018). Xiang et al. report that ac4C is mediated by NAT10 (N-acetyltransferase 10), while the deletion of NAT10 decreased the oocyte maturation rate in the mouse subject.

In males, spermatogenesis occurs throughout the entire life by maintaining the spermatogonial stem cells and this process is precisely regulated (Neto et al., 2016). After the proliferation of spermatogonia, they enter meiosis to become spermatocytes, which further differentiate into round spermatids through consecutive meiotic divisions. The round spermatids then undergo transformation to become spermatozoa, which are released into the seminiferous tubule lumen. Spermatozoa undergo further maturation in the epididymis. This process is regulated by hormones, pre-mRNA alternative splicing, non-coding RNA, epigenetic modifications, micro-environment, etc*.* (Neto et al., 2016). Non-obstructive azoospermia (NOA) is a crucial reason for male infertility, but the causes of ∼70% of NOA are still termed idiopathic NOA (iNOA). Tang et al. find that some males were diagnosed with iNOA in the clinic, but they had been fertile. To investigate, they test the mRNA profiling in the testicular tissues of these males and find the mRNA expression was altered compared to obstructive azoospermia. Wu et al. find that the deletion of SYMPK blocked spermatogenesis and led to infertility in mice because the pre-mRNA alternative splicing was disturbed. During post-testicular sperm maturation, there is a dynamic change process of non-coding RNA (Sharma et al., 2018) and a re-methylation process of the *Pgk-2, ApoA1*, and *Oct-3/4* loci (Ariel et al., 1994). These indicate that non-coding RNA and the re-methylation of genes are essential for sperm maturation. In this topic, Chadourne et al. report that *Topaz1* is important for spermatogenesis mediated by lncRNA. Chen et al. find that the global methylation in sperm from the testis was significantly different from sperm from the caput epididymis. The microenvironment is also important for spermatogenesis. For example, in the testis, hypoxia leads to abnormal spermatogenesis and infertility (Jankovic Velickovic and Stefanovic, 2014). Li et al. review the relationship between hypoxia, induced by environmental and pathological factors, and male infertility in humans and animals and discuss the potential mechanisms.

## Metabolic disorders have adverse effects on reproduction and offspring health

PCOS is a major cause of female infertility. For women with PCOS, ovarian function is reduced, resulting in anovulation and low oocyte competence. Studies in mice show that the global gene expression in ovaries and granulosa cells is altered by PCOS, including genes associated with oocyte meiosis (Palomba et al., 2017; Snider and Wood, 2019). Gao et al. demonstrate that PCOS leads to an increase in the expression of USP25 in granulosa cells, which regulates the proliferation and apoptosis by decreasing the expression of PI3K, AKT, and BCL2, and increasing the expression of *Bax*. Li et al. report that PCOS altered the transcriptional profiling in oocytes and cumulus cells compared with age-matched non-PCOS women.

Since the 1960s, researchers have been exploring the oocyte metabolome to identify those with the greatest potential to produce a successful pregnancy (Collado-Fernandez et al., 2012). Harris et al. find that, during folliculogenesis, glucose is utilized by intact follicles while pyruvate is the main metabolite consumed by oocytes during folliculogenesis (Harris et al., 2007; Harris et al., 2009; Collado-Fernandez et al., 2012). Amino acid is also crucial for follicular development, fertilization, and early embryo development (Hong and Lee, 2007). Liu et al. analyze the metabolome landscape during oocyte maturation and elucidate the metabolic pathway of polyunsaturated fatty acids regulating oocyte maturation (Li et al., 2020). The pivotal role of lipid metabolism during oocyte maturation is reviewed by Liu et al. PCOS may alter the metabolic profile in serum and follicular fluid, contributing to an important etiology of low oocyte competence. Liu et al. report that high levels of total cholesterol (TC), triglycerides (TG), and low density lipoprotein cholesterol (LDL-C) are associated with high oocyte retrieval, but obesity is associated with lower oocyte maturation rate, fertilization rate and good-quality embryo rate, as well as a poor live birth outcome for women with PCOS undergoing an unstimulated natural cycle. Huang et al. analyze the follicular fluid of PCOS women using Raman spectra, and find an association of these values with blastocyst rate and clinical pregnancy rates. When Raman spectra is matched with machine-learning algorithms, an accuracy of 90% and 74% in evaluating oocyte competence and clinical pregnancy of PCOS patients, respectively, can be achieved. Raman spectra are also used to predict male reproductive capacity, as reviewed by Zhang et al..

The WHO (World Health Organization) reported that diabetes mellitus and obesity are two of the most frequent metabolic diseases worldwide. (https://www.who.int/news-room/fact-sheets/detail/diabetes; https://www.who.int/news-room/fact-sheets/detail/obesity-and-overweight). Diabetes and obesity cause many complications in health and adverse effects on reproduction. For example, diabetes and obesity lead to ovarian inflammation and low oocyte quality (Snider and Wood, 2019). However, the mechanisms underlying ovarian dysfunction due to diabetes or obesity are not completely elucidated. Adamowski et a1. find that leptin signaling plays an important role in the activation of NOD-like receptor protein 3 (NLRP3) inflammasome in the ovaries of obese mice. Ge et al. report that the loss of PDK1 is a major cause of the abnormal maturation of oocytes in diabetic mice. Furthermore, the offspring of females with diabetes or obesity have a higher risk of chronic diseases in adulthood, such as cardiovascular diseases and metabolic disorders. Dong et al. report that the offspring of mothers with type 2 diabetes and gestational diabetes had a higher risk of malformations and death at birth.

## Effects of extra-environmental factors on reproduction

Environmental pollution is a great threat to public health, including reproductive health (Malott and Luderer, 2021; Liu et al., 2022). For example, heavy metals exposure has deleterious effects on gametogenesis, resulting in impaired development in early embryos, fetuses, and offspring (Rzymski et al., 2015; Bhardwaj et al., 2021). Exposure to air pollution, for instance, including particulate matter (PM), polychlorinated biphenyls (PCBs), sulfur dioxide (SO2), and nitrogen dioxide (NO2), leads to subfertility/infertility, low oocyte and sperm quality, and imbalanced endocrine function (Kampa and Castanas, 2008; Grippo et al., 2018). Nicotine, an important air pollutant from smoking, induces abnormal folliculogenesis and autophagy of ovarian cells at birth (Wang et al., 2018). Liu et al. report that the damage to early folliculogenesis induced by nicotine could be alleviated by high dosages of LH (luteinizing hormone) and FSH (follicle-stimulating hormone). Synthetic chemical compounds and some components of plants also have adverse effects on reproduction. The deleterious effects of Bisphenol A (BPA) and phthalates on germ cells and embryos are well known (Lehraiki et al., 2009; Rajkumar et al., 2022). Niu et al. demonstrate that hexestrol, a chemical compound used in livestock production and aquaculture, disturbs oocyte maturation and embryo development. Li et al. show that Aristolochic acid I reduces oocyte maturation, embryo development, and mitochondrial function of oocytes.

Assisted reproductive technologies (ARTs) are widely used in humans, domestic animals, and animal models. ART has become the most effective way to treat infertility/subfertility in humans, and more than 5 million ART babies have been born since 1978 when the first ART child was born in Great Britain. Thus, the safety of ARTs has to be considered. Clinical studies indicate that ART increases the risk of low birth weight, preterm, stillbirth, gestational diabetes, malformations in infants, and chronic diseases (Chen and Heilbronn, 2017; Wijs et al., 2021). The adverse effects of ART on offspring may start during the manipulations to obtain germ cells and early embryos. For example, exogenous hormones, used in ovarian stimulation reduce oocyte quality and embryo developmental potential (Marshall and Rivera, 2018). The culture medium cannot completely mimic the *in vivo* environment and may have adverse effects on germ cells and embryos. Oocyte freezing damages cellular organelles and reduces embryo developmental potential. Micromanipulations, such as ICSI, may also have deleterious effects on embryo development (Marshall and Rivera, 2018). Evidence from human and animal models has proved that epigenetic modifications, including DNA methylation, histone modifications, micro-RNAs, RNA modifications, and chromosome structure, are prone to be disturbed by ARTs during the maturation of germ cells and early embryo development (Menezo and Elder, 2020) that could lead to aberrant fetal development (Liang et al., 2013; Saenz-de-Juano et al., 2019). For example, Xu et al. report that cryopreservation of sperm altered the miRNA profile, which may play a role in the low blastocyst rate after fertilization in mice.

It is a great challenge to minimize the adverse effects of ARTs on germ cells, embryos, and offspring. Clinicians have used low-dosage exogenous hormones and gonadotropin-releasing hormone (GnRH) antagonist-based ovarian stimulation protocol to reduce their adverse effects (Wang et al., 2021). Chen et al. report that a tip pipette, combined with a piezoelectric-assisted manipulator, increases the survival rate of oocytes and embryos after mRNA microinjection in mice. Chu et al. show that vitamin C prevents the active DNA methylation of early mouse embryos that takes place during *in vitro* culture. Hou et al. show that minocycline hydrochloride alleviates the adverse effects of the medium on early embryos by inhibiting *Parp1* (Poly (ADP-ribose) polymerase-1) in mice. Tang et al. present evidence that glycine and melatonin could improve the embryo development produced by vitrified oocytes of mice. Hao et al. report that making a small hole in the zona pellucida of a morula stage embryo improves the hatching rates in mice. More studies are still required to reduce the deleterious effects of ARTs on germ cells, embryos, and offspring.

## Uterine endometrium and implantation

Naturally, the incidence of successful pregnancies is no more than 30% in each menstrual cycle. Approximately 75% of the lost conceptions are caused by implantation failure (Zhang et al., 2013). For successful implantation to take place, the blastocysts have to acquire the implantation competency and the uterine stroma needs to differentiate into epithelial-like secretory decidual cells, known as decidualization, which is essential for embryonic growth and invasion. Decidualization is also important for the semi-allogenic embryo to escape from the maternal immunological responses (Zhang et al., 2013). These two events are hierarchically regulated by many factors, including estrogen and progesterone (Zhang et al., 2013). Zhu et al. report that monosodium urate enhances the transformation of uterine stromal cells into decidual cells, and Zhu et al. find that a higher expression of insulin-like growth factor 2 mRNA-binding protein 3 (IGF2BP3) may induce spontaneous abortion impairing decidualization. The crosstalk between mother and fetus is also regulated by the chemokines, as reviewed by Zhang et al.

## Summary

In this Research Topic, a total of 31 papers were accepted by reviewers and editors. Of these papers, seven contribute to the understanding of gametogenesis; nine explore the effects and the possible mechanisms of metabolic disorders including PCOS, diabetes, obesity, and aging on germ cells and offspring; four provide new knowledge on the adverse effects of environmental pollution on germ cells and embryos, and how to alleviate the deleterious effects; seven investigate the adverse effects of ART on germ cells and embryos and explore how to reduce them; and, finally, four are focused on the maternal-fetal interface during implantation. These papers greatly contribute to our understanding of the mechanisms underlying the effect the intra- and extra-environment have on reproduction and encourage more studies on this topic in the future.
